# Treatment seeking behaviour and associated factors among adults with high blood pressure from three selected states in Nigeria

**DOI:** 10.1371/journal.pgph.0002949

**Published:** 2024-04-17

**Authors:** Eniola Bamgboye, Abiola Ayoyemi, Mobolaji Modinat Salawu, Joshua Odunayo Akinyemi, Okechukwu Samuel Ogah, Uzoamaka Alice Uja, Rabiu Ibrahim Jalo, Oyediran Oyewole, Mahmoud Sani, IkeOluwapo Oyeneye Ajayi

**Affiliations:** 1 Faculty of Public Health, Department of Epidemiology and Medical Statistics, College of Medicine, University of Ibadan, Ibadan, Nigeria; 2 Faculty of Clinical Sciences, Department of Medicine, College of Medicine, University of Ibadan, Ibadan, Nigeria; 3 Department of Public Health, Ministry of Health, Abia State, Umuahia, Nigeria; 4 Department of Community Medicine, Bayero University Kano, Kano, Nigeria; 5 Faculty of Public Health, Department of Health Promotion and Education, College of Medicine, University of Ibadan, Ibadan, Nigeria; South African Medical Research Council, SOUTH AFRICA

## Abstract

Management of hypertension is challenging in multi-cultural and multi-ethnic sub-Saharan African countries like Nigeria. This diversity calls for multi-dimensional interventional approaches for hypertension control. This study assessed the treatment seeking behaviour and associated factors among adults with high blood pressure from three ethnic groups in Nigeria. A cross-sectional study was conducted among 762 adults with high blood pressure from three purposively selected States representing the three main tribes in Nigeria. Using a multistage stratified sampling technique, five communities were selected from two Local Government Areas (LGAs) stratified into urban and rural LGAs in each State. All consenting respondents in each community were consecutively screened for hypertension and recruited. A pretested interviewer-administered questionnaire was used to obtain information on socio-demographic characteristics, treatment seeking behaviour and factors affecting their choice. Data were summarized using descriptive statistics. Relationship between individual, health-related factors and treatment seeking behaviour, as well as the predictors were assessed using a binary logistic regression. at p<0.05 Participants’ mean age was 55.4 ± 16.6 years, 63.0% were females and most were Igbo speaking (39.9%). About half (368, 48.3%) were unaware of their status. Of those aware, most (58.9%) went to hospital upon diagnosis of hypertension while some sought advice from health care professionals (28.5%) mostly Hausas, others either went to chemists (6.2%) or did nothing (5.1%), predominantly Yorubas. Significant predictors of orthodox treatment seeking practices for hypertension were female gender [(AOR = 2.60; 95%CI (1.18–5.71)], availability of medicine and personnel [(AOR = 8.7; 95%CI (4.15–18.3)] and perceived good quality of care [(AOR = 4.88; 95%CI (1.81–13.1)]. Orthodox treatment was the common choice among adults with high blood pressure. To further encourage patronage of orthodox treatment, the health facilities should be adequately equipped with medications and trained personnel to improve the quality of care. Targeted education on continuous practice of orthodox treatment is recommended.

## Introduction

Worldwide, hypertension (HTN) is the leading cause of cardiovascular disease [1,2]. Often termed the "silent killer," hypertension is mostly asymptomatic, leading to a lack of awareness among the majority of affected individuals. Consequently, many remain unmanaged until complications arise, emphasizing the importance of timely medical attention. Effective control of hypertension involves proactive treatment-seeking behavior, a key factor in preventing complications [3–5].

Treatment seeking behaviour encompasses actions taken by individuals who recognize they have a health condition or feel unwell enough to seek an appropriate remedy from a trained physician in a healthcare facility [6]. Proper management and control of hypertension, have been attributed to pattern of treatment seeking behaviour [7,8].

Charkraboty et al. [9] in their study among hypertensives in India found that 80% of hypertensives received care from non-public facilities including informal health care providers. Furthermore, a similar study carried out by Musinguzi et.al. [10] in Uganda found that self-medication, use of over-the-counter antihypertensive drugs with or without prescription and herbal remedies were the common treatment practices. In Nigeria, researchers have also reported varying treatment seeking behaviors among hypertensives. Public health facilities and patent medicine vendors were the major first point of call while others still visited traditional medicine peddlers [11–13].

Researchers in low- and middle- income countries have documented various factors influencing treatment seeking behaviors and these are usually influenced by individual, societal, environmental and health system factors. Some of these include desire to feel better, fulfil family responsibilities, social status, availability of services, cost of treatment, distance to health facilities, lack of trust in the healthcare system, and perceived severity of hypertension [9,14–18]. In Nigeria, similar factors were also reported such as quick accessibility, reduced waiting time, cost of treatment, distance to health facilities and lack of freedom to make choice on the type of care [11–13].

Recent reviews have reported a continuous rise in cases of hypertension in Nigeria [18–21] with antecedent poor management and control that might be related to pattern of treatment seeking practice which can also be influenced by cultural norms. Due to varying cultural practices interventions to control hypertension need to be context specific and based on evidence. Hence, this study was conducted to provide information on the treatment seeking behavior of adult hypertensive patients and factors associated in selected ethno-cultural representative states in Nigeria in order to inform policy decisions and improve hypertension management practices.

## Methodology

The data presented in this paper were extracted from a baseline survey conducted as part of a cluster randomized control trial to assess the effectiveness of a community-based support for hypertension control in three selected states in Nigeria, namely Abia, Kano and Oyo. The study protocol has been published elsewhere [22]. The three states are located in South Western(Oyo state), South Eastern (Abia state) and North Western Nigeria. They represent the three main ethnic groups found in Nigeria and have a relatively high prevalence of hypertension among adults ranging from 28.5%-33% [23].

In these states, hypertension is being managed at all levels of health care system including tertiary, secondary and primary health care centers. Other sources of health care in the communities include patient medicine vendors and itinerary drug hawkers some of which peddle prescription drugs including anti-hypertensive drugs [24–26].

The baseline study was a community-based cross-sectional survey which utilized a multistage cluster sampling technique to select two local government areas (1 urban and 1 rural) in each state. In each LGA, all communities were identified and 5 were randomly selected through balloting with consideration for proportion to population size. A total of 1704 community members aged above 18 residing in the communities for at least one year had their blood pressure measured irrespective of history of high blood pressure levels or hypertension. Out of 1704 community members, 762 were found to have high blood pressure as at the time of the survey. All the 762 adults with high blood pressure constituted the sample size for this study.

### Data collection

A validated, pretested semi-structured, interviewer-administered questionnaire was used to collect data. It consisted of four sections; Socio-demographic characteristics, awareness of hypertension, treatment seeking practices of hypertension and plausible factors influencing treatment seeking practices of hypertension. The study instrument was translated to local languages–Hausa, Igbo and Yoruba languages Content and face validity of the instruments was conducted by 3 experts–a cardiologist (OSO), epidemiologist (IOA)and health promotion specialist (OO)

#### Blood pressure measurement

A digital blood pressure monitor (Omron M3) with appropriate cuff size was used to measure blood pressure. Three measurements were taken at least 2 minutes interval on the left arm. The mean of the last two readings was determined. Hypertension was defined as systolic blood pressure (SBP) ≥140 mmHg and/or diastolic blood pressure (DBP) ≥90 mmHg (1). Participants with such blood pressure readings were recruited for the study [2].

#### Treatment seeking behavior

The treatment seeking behaviour for hypertension was categorized into orthodox treatment and unorthodox treatment. Orthodox treatment included seeking advice from medical professionals and visiting a health facility while unorthodox treatment included visiting a chemist / pharmacy, patent medicine vendor, spiritual leader, use of contemporary/alternative medicine as well as those that took no action.

Data were analyzed using STATA version 17. Data were summarized using descriptive statistics: such as mean, standard deviation for continuous variables, and proportions for categorical variables. Frequency tables and charts were used to illustrate the results. The treatment seeking behaviour of the hypertensive patients were presented as percentages before being categorized into orthodox and unorthodox treatment behaviour. Inferential statistics was performed using Chi- squared test and binary logistic regression at p<0.05. Chi-square test was used to test significance of association between the outcome variable: treatment seeking practices and explanatory variables including socio demographic and health system factors. The significant variables on bivariate analysis at p<0.1 were selected for binary logistic regression, which was used to determine predictors of treatment seeking practices for HTN.

### Ethical considerations

Ethical clearance for this study was obtained from the National Health Research Ethics Committee of Nigeria (NHREC) of the Federal Ministry of Health Nigeria-NHREC/05/01/2008a and the University of Ibadan/University College Hospital (UI/UCH) Ethics Committee–UI/UCH/19/0448. In addition, written informed consent as well as verbal informed consent (for those who couldn’t write) was also obtained from all respondents involved in the study.

## Results

A total of 762 respondents who were found to have blood pressure at baseline were recruited in all the three States, with the highest proportion from Abia 304 (39.8%) followed by Kano 283 (37.1%) and the least from Oyo State, 175 (22.9%). (Table 1) Mean age of the respodents was 58.9 ±15.8 years in Abia, 54.1 ± 16.8 years in Oyo and 52.5 ±15.8 years in Kano State (F = 12.49, p <0.01). Majority were females (63.0%) which was similar in Oyo (67.4%) and Abia (70.7%) State unlike Kano State where half, 51.9%, were females. Majority of the respondents with high blood pressure recruited were married (80.7%), earned less than $65 monthly (65.5%) and were traders (40.8%) (Table 1).

**Table 1:** Frequency distribution of the socio-demographic characteristics of adults with high blood pressure in three selected states in Nigeria(N = 762).

	Total (N = 762) n (%)	Oyo (n = 175) n (%)	Abia (n = 304) n (%)	Kano (n = 283) n (%)	p-value
**Age group (years)**					
< 40 years	131(17.2)	38(21.7)	39(12.8)	54(19.1)	
40–59 years	281(36.9)	67(38.3)	97(31.9)	117(41.3)	0.001
60 years and above	350(45.9)	70(40.0)	168(55.3)	112(39.6)	
**Gender**					
Male	282(37.0)	57(32.6)	89(29.3)	136(48.1)	<0.001
Female	480(63.0)	118(67.4)	215(70.7)	147(51.9)	
**Marital Status**					
Currently Married	615(80.7)	118(67.4)	260(85.5)	237(83.8)	
Divorced/Separated/Widowed	114(15.0)	46(26.3)	28(9.2)	40(14.1)	<0.001
Never Married	33(4.3)	11(6.3)	16(5.3)	6(2.1)	
**Education**					
No formal education	258(33.9)	45(25.7)	52(17.1)	161(56.9)	
Primary	197(25.9)	41(23.4)	115(37.8)	41(14.5)	<0.001
Secondary	192(25.2)	67(38.3)	80(26.3)	45(15.9)	
Tertiary	115(15.1)	22(12.6)	57(18.8)	36(12.7)	
**Monthly income (USD$)**					
<65	431(65.5)	98(60.1)	171(75.3)	162(60.5)	
≥65	227(34.5)	65(39.9)	56(24.7)	106(39.6)	0.001
**Domicile**					
Rural	366(48.0)	42(24.0)	160(52.6)	164(58.0)	
Urban	396(52.0)	133(76.0)	144(47.4)	119(42.0)	<0.001
**Religion**					
Christianity	387(50.8)	82(46.9)	304(100.0)	1(0.4)	
Islam	372(48.8)	91(52.0)	0.0	281(99.3	<0.001
Traditional	3(0.4)	2(1.1)	0.0	1(0.4)	
**Ethnicity**					
Hausa	282(37.0)	0.0	0.0	282(99.7)	
Igbo	315(41.3)	11(6.3)	303(99.7)	1(0.3)	<0.001
Yoruba	165(21.7)	164(93.7)	1(0.3)	0.0	
**Occupation**					
Trading/Buisness	311(40.8)	124(70.9)	91(29.9)	96(33.2)	
Not working	190(24.9)	26(14.9)	61(20.1)	103(36.4)	
Farming	180(23.6)	11(6.3)	128(40.5)	46(16.3)	<0.001
Employed	81(10.6)	14(8.0)	29(9.5)	38(13.4)	

About half (48.3%) of the respondents with high blood pressure were unaware and this was highest in Kano State (60.4%), followed by Oyo State (49.1%) and lowest in Abia State (36.5%). (Table 2). Concerning the respondents’ general health seeking behavior, about half of all the respondents (367, 48.2%) never visited a health facility in the last one year which was highest in Oyo State (58.3%), followed by Kano State (55.5%), and lowest in Abia State (35.6%). Only 107 (14.0%) of the adults with high blood pressure visited a health care provider monthly. (Table 2) Majority of the respondents (47.5%) treated themselves at home whenever they fall sick, while about 20.0% visited a primary health center, 7.9% consulted PPMVs/pharmacies and 8.7% claimed they never felt sick. Only about 10.0% of the respondents had any other disease that required daily medication with higher proportions in Oyo (27, 15.4%) and Abia State (46, 15.1%) (Table 2).

**Table 2:** Frequency distribution of selected characteristics of adults with high blood pressure in selected states in Nigeria(n = 762).

	Oyo (n = 175) n (%)	Abia (n = 304) n (%)	Kano (n = 283) n (%)	Total (N = 762) n (%)
**Prior Knowledge of Hypertension**				
Known	89(50.9)	193(63.5)	112(39.6)	394(51.7)
Unknown	86(49.1)	111(36.5)	171(60.4)	368(48.3)
**Comorbidity Present**				
Yes	27(15.4)	46(15.1)	12(4.2)	85(11.2)
No	148(84.6)	258(84.9)	271(95.8)	677(88.8)
**Frequency of Visiting a Health Care Provider**				
Monthly	10(5.7)	60(19.8)	37(13.1)	107(14.1)
At least once in six months	37(21.1)	79(26.1)	41(14.5)	157(20.6)
Once in the last one year	26(14.9)	56(18.5)	48(16.9)	130(17.1)
Never in the last one year	102(58.3)	108(35.6)	157(55.5)	367(48.2)
**First step when sick**				
Treat at home with drugs bought	84(61.3)	55(26.2)	149(57.3)	288(47.5)
Visit nearest PHC	8(5.8)	63(30.0)	47(18.1)	118(19.4)
Consult PPMVs/Pharmacy	3(2.2)	30(14.3)	15(5.8)	48(7.9)
Visit other health facility	7(5.1)	23(10.9)	11(4.2)	41(6.7)
Consult health worker in Community	3(2.2)	21(10.0)	11(4.2)	35(5.8)
Use Herbs	13(9.5)	0(0.0)	1(0.4)	14(2.1)
Rest	8(5.8)	0(0.0)	0(0.0)	8(1.3)
Faith Based Organizations	2(1.5)	0(0.0)	0(0.0)	2(0.3)
Never fall sick	9(6.6)	18(8.6)	26(10.0)	53(8.7)
**How do you rate your health presently**				
Excellent	19(10.9)	9(3.0)	9(3.2)	37(4.9)
Very Good	51(29.1)	49(16.2)	56(19.8)	156(20.5)
Good	70(40.0)	144(47.5)	167(59.0)	381(50.1)
Fair	30(17.1)	76(25.1)	47(16.6)	153(20.1)
Poor	5(2.9)	25(8.2)	4(1.4)	34(4.4)

Of the 762 adults with high blood pressure, 391(51.3%) knew their status before the survey, and among these, majority (82.9%) were diagnosed as hypertensives at the hospital and this was similar across all the states. However, about 6% were diagnosed at outreach/research centers. (Table 3) Regarding the treatment seeking behaviour, most of the known respondents with high blood pressure sought help within seven (7) days of being diagnosed hypertensive which was seen to be highest in Kano State (92.0%) while about 10% did so after about one month and this was highest in Abia State. (Table 3) However, concerning blood pressure check, respondents in Abia State had the highest proportion who had their blood pressure checked within the last one month (49.2%) and between a month and three month (31.2%) prior to the survey. More respondents checked their blood pressure more than 3 months prior to the survey in Kano (36.0%), followed by Oyo (29.6%) and Abia (12.7%). However, only 7.8% of the known respondents with high blood pressure had never checked their blood pressure within the last one year prior to the survey and this was seen to be higher among respondents from Oyo State. Furthermore, about a quarter of the adults with known high blood pressure had their blood pressure controlled prior to the survey. This was highest among respondents in Abia (48.2%), followed by Oyo (44.9%) and least in Kano (23.2%) (Table 3).

**Table 3:** Selected characteristics of known adults with high blood pressure in selected states in Nigeria (n = 394).

	Oyo (n = 89) n (%)	Abia (n = 193) n (%)	Kano (n = 112) n (%)	Total (n = 394) n (%)
Where diagnosis of hypertension was made*				
Hospital	54(60.7)	162(85.3)	108(96.4)	324(82.9)
Medicine Seller/Chemist	11(12.3)	23(12.1)	0(0.0)	34(8.7)
Outreach	16(17.9)	3(1.6)	3(2.7)	22(2.8)
Researchers	8(8.9)	2(1.0)	1(0.9)	11(2.8)
Duration between diagnosis and help seeking *				
Within 7 days	68(86.1)	132(72.5)	103(92.0)	303(81.2)
Within 4 weeks	3(3.8)	21(11.5)	9(8.0)	33(8.9)
After One Month	8(10.1)	29(15.9)	0(0.0)	37(9.9)
Last time high blood pressure was measured by health professional*				
Less than a month	15(21.1)	93(49.2)	32(28.8)	140(37.7)
Between 1 and 3 months	25(35.2)	59(31.2)	33(29.7)	117(31.5)
More than 3 months ago	21(29.6)	24(12.7)	40(36.0)	85(22.9)
Never checked in last 1 year	10(14.1)	13(6.9)	6(5.4)	29(7.8)
**Blood pressure status**				
Controlled	40(44.9)	93(48.2)	26(23.2)	159(40.4)
Uncontrolled	49(55.1)	100(51.8)	86(76.8)	235(59.6)

**Fig 1:**
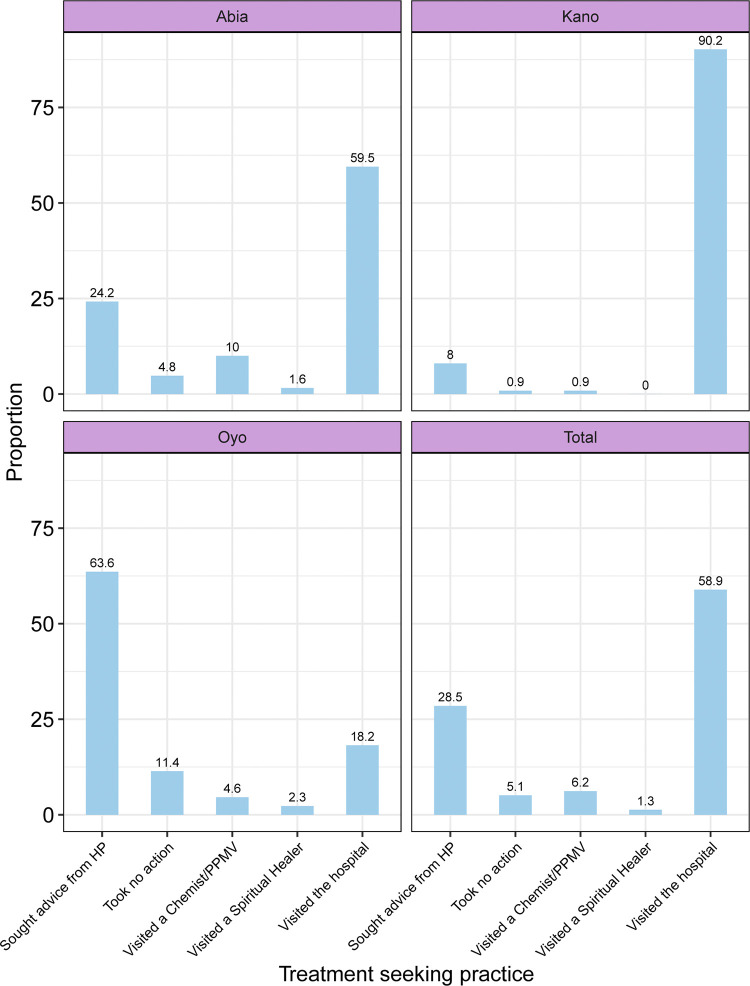
Pattern of treatment-seeking behaviour of the adults with known high blood pressure in selected states in Nigeria.

Fig 1 presents the details of the treatment-seeking behaviour of the adults with known high blood pressure. About 60% of the adults with known high blood pressure visited a hospital after being diagnosed with hypertension and this was highest in Kano State (90.2%) and least in Oyo State (18.2%) while about a third sought advice from health care providers informally which was the common practice among the respondents in Oyo State (63.6%). Others went to the chemist/PPMVs (6.2%), took no action (5.1%) or went to a spiritual leader (1.3%). Of the respondents who went to chemist/PPMVs, a higher proportion were from Abia State (10.0%) while Oyo state had the highest proportion of those who took no action (11.4%) or went to a spiritual leader (2.3%) In terms of type of treatment seeking behaviour, overall, a vast majority (87.4%) of the respondents practiced orthodox methods following diagnosis of hypertension. Orthodox treatment was practiced by almost all respondents in Kano (98.2%), 83.7% in Abia and 81.8% in Oyo State.

Table 4 shows the relationship between selected socio-demographic characteristics and choice of treatment seeking behavior. Result shows that a higher proportion of those who practiced orthodox treatment were aged 60 years and above (90.8%), had no formal education (93.5%), were Hausas (98.2%) and unemployed (95.6%). Moreover, a slightly higher proportion of those earning less than $65 monthly practiced unorthodox treatment (14.0%) as compared to those earning above $65 (10.9%) while a similar proportion of those living in rural and urban residence practiced unorthodox treatment. Petty traders/business men/women and farmers practiced unorthodox treatment as compared to other occupational categories. However, the difference in these proportions were found not to be statistically significant(p>0.05). Hausa speaking(p = 0.00) and those living in Kano (p = 0.00) constitute respondents who had a statistically significant higher proportion using orthodox treatment. (p<0.05).

**Table 4:** Relationship between selected characteristics and choice of treatment sought among adults with high blood pressure.

	Treatment seeking practices					
	Unorthodox (n = 49)	Orthodox (n = 341)	cOR	95%CI	aOR	95%CI
**Age group (years)**						
Less than 40	8(16.0)	42(84.0)	1		1	
40–59	23(15.9)	121(84.0)	1.00	0.4–2.4	0.93	0.31–2.74
60 years and above	18(9.2)	178(90.8)	1.88	0.7–4.6	1.89	0.56–6.48
**Gender**						
Male	18(15.5)	98(84.5)	1		1	
Female	31(11.3)	243(88.7)	1.43	0.76–2.69	2.83	1.23–6.51*
**Education**						
No formal education	7(6.5)	101(93.5)	1		1	
Primary	17(14.8)	98(85.2)	0.40	0.15–1.0	1.39	0.44–4.41
Secondary	16(16.5)	81(83.5)	0.35	0.13–0.89*	1.50	0.42–5.40
Tertiary	9(12.9)	61(87.1)	0.46	0.16–1.32	1.96	0.49–7.81
**Income (USD$)**						
<65	30(14.0)	184(86.0)	1			
≥(65	13(10.9)	196(89.1)	1.32	0.6–2.6		
**Domicile**						
Rural	233(68.1)	109(31.9)	1			
Urban	263(69.9)	113(30.1)	0.79	0.43–1.47		
**Ethnicity**						
Hausa	2(1.8)	109(98.2)	1			
Igbo	31(16.1)	162(83.9)	0.09	0.02–0.48		
Yoruba	10(18.6)	70(81.4)	0.08	0.01–0.35		
**Occupation**						
Employed	6(14.3)	36(85.7)	1			
Farming	14(15.9)	74(84.1)	0.88	0.31–2.48		
Trading/Business	25(14.7)	145(85.3)	1.72	0.3–7.46		
Not Working	4(4.4)	86(95.6)	0.86	0.32–2.29		
**State**						
Oyo	16(18.2)	72(81.8)	1		1	
Abia	31(16.3)	159(83.7)	1.13	0.5–2.2	1.15	0.51–2.57
Kano	2(1.8)	110(98.2)	12.2	2.7–54.7	13.1	2.57–66.5*
**Perceived quality of care**						
No	43(15.1)	242(84.9)	1		1	
Yes	42(56.8)	32(43.2)	2.9	1.2–7.1	5.4	1.96–14.8*
**Availability of Medicine, Personnel and Diagnostic Supplies**						
No	31(32.6)	64(67.4)	1		1	
Yes	18(6.1)	227(94.0)	7.45	3.9–14.1	10.3	4.9–21.8*

cOR: Curde Odds Ratio; aOR: Adjusted Odds Ratio

*significant predictors.

When asked the reasons for choice of treatment seeking behaviour, a higher proportion of those who reported the availability of medicine, personnel and diagnostic supplies influenced their choice, practiced orthodox treatment (94.0%) as compared to those who felt these services did not influence their choice of treatment seeking (67.4%). However, a higher proportion of respondents who perceived quality of care (56.8%) as influencing their choice of treatment practiced unorthodox treatment. (cOR:2.9 95%CI: 1.2–7.1)

Binary logistic regression analysis showed that gender, state of residence, perceived quality of care and availability of medicine, personnel and diagnostic supplies were independent predictors of seeking orthodox treatment. Females were about 3 times more likely to seek/practice orthodox treatment compared with males (AOR:2.6; 95% CI:1.2–5.7) while respondents in Kano State were about 14 times more likely (AOR: 13.5; 95%CI: 2.7–67.1) to practice orthodox treatment than those living in Oyo State. Respondents who were influenced by availability of medicine, personnel and diagnostic supplies were about 9 times more likely to practice orthodox treatment (AOR: 8.7; 95%CI:4.2–18.3) while those who perceived quality of care as good were about 5 times more likely to seek orthodox treatment (AOR: 4.88; 95%CI:1.8–13.1) (Table 4).

## Discussion

This study was conducted to assess the treatment seeking behaviour and associated factors among adults with high blood pressure recruited as part of a base line longitudinal study in selected states representing the three major ethnic groups in Nigeria.

We found out that about 8 out of 10 known adults with high blood pressure visited health care facilities or sought advice from health care providers following diagnosis of being hypertensive which is similar to a study done by Osamor [12] on the health care seeking for hypertension in South West Nigeria where 63.4% of the respondents reported that they sought care from a hospital/ health facility. This finding could be due to the fact that the majority of the respondents were diagnosed to be hypertensive at the hospital or when health care workers visited them on outreaches. However, this did not translate to the respondents seeking orthodox treatment regularly, as about a third of the hypertensive had not had their blood pressure checked by a healthcare provider in the last three months preceding the survey and a sixth had their blood pressure uncontrolled.

Hitherto, a considerable number of adults with high blood pressure still visited patent medicine vendor, traditional healers or contemporary and alternative medicines, while some who visited hospitals still took herbal or traditional medicines. Based on our findings we could not deduce that these adults with high blood pressure adhere to seeking treatment from only hospitals or health care providers. This multiple choice of treatment seeking behaviour could be have a negative implication on hypertension control. The negative impact of diverse treatment-seeking behaviors on hypertension control became apparent, with only about a quarter achieving blood pressure control despite receiving treatment.

Availability of medicines, personnel and diagnostic supplies as well as patient’s perception about their provider’s abilities were found to be associated with patient’s choice of seeking orthodox treatment for hypertension. This corroborates a study conducted by Musinguzi et al [10] which also reported availability of service as one of the reasons which influence hypertensive patients’ treatment-seeking behaviour. This finding shows the importance of health system factors as a major driver of seeking orthodox practices.

In addition, findings revealed that about half of the adults with high blood pressure were unaware of their status and this was highest in Kano State, Northern—Nigeria. This is not surprising as Odili et al [23], in their national study found that about half (52%) of the hypertensives in North Western Nigeria were not aware though with higher proportions in North Central and North Eastern parts of the Country. In other developing countries similar findings were reported by Prenissel et al [4] in a study in northern India, where 55% of individuals were not aware of their hypertensive status. Unawareness of hypertensive status has an implication on development of complications which could lead to major cardiovascular diseases such as stroke and thus increase the burden in these countries if actions are not taken.

The authors acknowledge that is study was limited by the cross-sectional nature which relied on self-reported treatment seeking behavior of the adults with high blood pressure as this could be influenced by social desirability bias. In addition, the overlap between the use of orthodox and unorthodox treatment practices could not be fully explored due to the cross-sectional nature of this study which could have been done with a follow-up study of each respondents treatment seeking practice. Despite these limitations, the study was still able to highlight that most adults with known high blood pressure do not comply with regular visit to health care providers(orthodox treatment practices) and this could impart on the control of hypertension in the country.

In conclusion, adults with high blood pressure in this study practiced orthodox treatment as first choice of treatment following diagnosis. Patients’ perception about health care workers competence and availability of resources were the major influencers of this decision. However, control of high blood pressure was not optimal due to inconsistencies in orthodox treatment seeking practices. People with high blood pressure need to be encouraged to continue to practice orthodox treatment throughout their life course of hypertension management. In addition, community programs or campaigns for regular blood pressure screening needs to be instituted to improve awareness of status.

## Supporting information

S1 Data Hypertension treatment seeking behaviour data. (DTA)
